# Use of Fructosamine for Glycemic Monitoring in Patients With Sickle Cell Disease and Diabetes: A Systematic Review

**DOI:** 10.7759/cureus.89130

**Published:** 2025-07-31

**Authors:** Mohammed Gaffar Mohammed, Nada A Alsiddig, Lobna A Ibrahim Hag

**Affiliations:** 1 Internal Medicine, Dr. Sulaiman Al-Habib Hospital Al Suwaidi, Riyadh, SAU; 2 Medicine and Surgery, Omdurman Islamic University, Omdurman, SDN

**Keywords:** fructosamine and haemoglobin a1c in sickle cell, fructosamine in diabetes, fructosamine in diabetes with sickle cell, fructosamine in sickle cell patient, hyperglycemia and fructosamine, sickle cell anemia

## Abstract

Sickle cell disease is characterized by various forms of hemoglobin that interfere with hemoglobin A1c (HbA1c) testing, which is commonly used to diagnose and monitor diabetes. This interference puts patients with sickle cell disease at risk of inaccurate monitoring and misdiagnosis due to improperly planned HbA1c testing. Despite awareness of these issues, there is still disagreement regarding the most appropriate method of measuring HbA1c in patients with sickle cell disease, along with a lack of clear guidance on using fructosamine as an alternative marker in patients with diabetes and sickle cell disease. We employed a systematic review technique to assess the efficacy of fructosamine in diagnosing and monitoring diabetes in patients with sickle cell disease. The study showed that fructosamine is highly associated with fasting blood glucose levels in patients with sickle cell trait, and even more so in those with sickle cell disease and poorly controlled diabetes. It also demonstrated a fair association with HbA1c, as measured by some of the recommended instruments for use in patients with sickle cell disease. These results suggest that fructosamine can play a valuable role in managing diabetes in patients with sickle cell trait and disease, along with HbA1c, as HbA1c is widely used to monitor diabetes, even in patients with diseases that might affect the accuracy of HbA1c measurement.

## Introduction and background

Sickle cell disease represents the most prevalent inherited structural defect in the production of hemoglobin molecules [1]. According to the World Health Organization, its occurrence is particularly significant in equatorial Africa, where prevalence ranges from 10% to 40% [2]. Sickle cell disease is also relatively common in Saudi Arabia. Approximately 4.2% of the Saudi population is a carrier of the sickle cell gene, while 0.26% have the disease. This prevalence varies across regions, with the highest carrier rate found in the Eastern Province (17%), where 1.2% of the population is affected [3].

Due to the rising rates of diabetes in Saudi Arabia, where 23.1% of the population is diabetic [4], there is a growing likelihood of encountering patients with both diabetes and sickle cell disease, especially in regions where the latter is common. One of the most commonly used tools to diagnose and monitor such patients is the hemoglobin A1c or HbA1c test.

HbA1c quantifies the glycated hemoglobin in the bloodstream. Since the typical lifespan of red blood cells is approximately 120 days, this test reflects average blood glucose regulation over the past three months [5]. This characteristic has established HbA1c as one of the scientific benchmarks for evaluating diabetes management, leading to its incorporation in nearly all guidelines for diabetes monitoring and diagnosis [6,7].

However, in individuals with sickle cell disease, the structure of hemoglobin interferes with certain HbA1c measurement methods, adversely affecting the test's accuracy [8]. As a result, an HbA1c test may give an inaccurate picture of glycemic control by either undervaluing or overvaluing the average blood glucose levels [9,10]. The American Diabetes Association (ADA) acknowledges this limitation due to the discrepancy between actual glucose levels and measured HbA1c in conditions that influence red blood cell turnover, such as hemolytic anemias (e.g., sickle cell disease) [11]. The National Glycohemoglobin Standardization Program (NGSP), which is responsible for standardizing HbA1c results, highlights the methods that are unreliable in the presence of sickle hemoglobin [8-12].

To avoid laboratory errors in such patients, the ADA recommends using only NGSP-certified HbA1c methods and, when necessary, applying plasma glucose criteria for diagnosis [11]. Additionally, because patients with sickle cell disease typically experience reduced red blood cell lifespans due to chronic hemolysis and often require repeated transfusions, measurement of HbA1c becomes even more unreliable.

Fructosamine offers a valuable alternative in such cases. It reflects glycated serum proteins over a shorter period (two to three weeks) and is unaffected by the structure of hemoglobin or the turnover of red blood cells. Therefore, it serves as a practical marker for monitoring glycemic control in patients with sickle cell disease [13].

This review aims to evaluate the effectiveness of fructosamine in diagnosing and monitoring diabetes in individuals with sickle cell disease compared to fasting glucose and HbA1c, with the goal of providing evidence-based recommendations for physicians in regions with high prevalence of sickle cell disease.

## Review

Methods

A literature review was carried out to study the use of fructosamine for diagnosing and monitoring diabetes in patients with sickle cell disease, as well as to compare its accuracy with fasting blood glucose and HbA1c. The complete inclusion and exclusion criteria are summarized in Table 1.

**Table 1:** Inclusion and exclusion criteria for the selection of studies

Category	Inclusion criteria	Exclusion criteria
Population	Patients diagnosed with both diabetes mellitus and sickle cell disease	Studies involving only diabetic patients without sickle cell disease, or fructosamine
Intervention	Use of fructosamine as a glycemic monitoring tool compared with HbA1c, fasting blood glucose, and random blood glucose	Studies not assessing or reporting the use of fructosamine
Comparator	Correlation between fructosamine and HbA1c or glucose	Studies that compared fructosamine to biomarkers other than HbA1c or blood glucose
Outcome	Reported at least one of the following: glycemic control measured by fructosamine, correlation between fructosamine and HbA1c or glucose	Studies not reporting glycemic outcomes related to fructosamine
Study type	Randomized controlled trials, cohort studies, case-control studies, cross-sectional studies, and systematic reviews	Case reports, editorials, and non-human cohort studies
Language	Published in English	Non-English publications
Time frame	Published between 2000 and 2022	Any study done before 2000 or after 2022
Availability	Studies with accessible data	Studies with inaccessible data

Boolean operators were used to focus on the most relevant papers for data selection. The search keywords included: “fructosamine and diabetes and sickle cell” and “HbA1c and sickle cell and diabetes.”

Using the Preferred Reporting Items for Systematic reviews and Meta-Analyses (PRISMA) flowchart [14], the PubMed and Embase databases were searched to identify 145 papers. Sixteen papers were removed due to duplication, leaving 129 papers for screening. Abstract reviews were used to screen the 129 papers, with 106 being rejected. The remaining 23 papers were further evaluated by a second peer reviewer, and 13 studies were excluded as they did not report fructosamine, as per the exclusion criteria.

Two additional studies were excluded after review by an independent reviewer: one was a systematic review proposal, and the other was not freely accessible. The main ideas from the final selected eight papers were documented in a matrix to facilitate analysis, peer review, and synthesis.

Results

**Figure 1:**
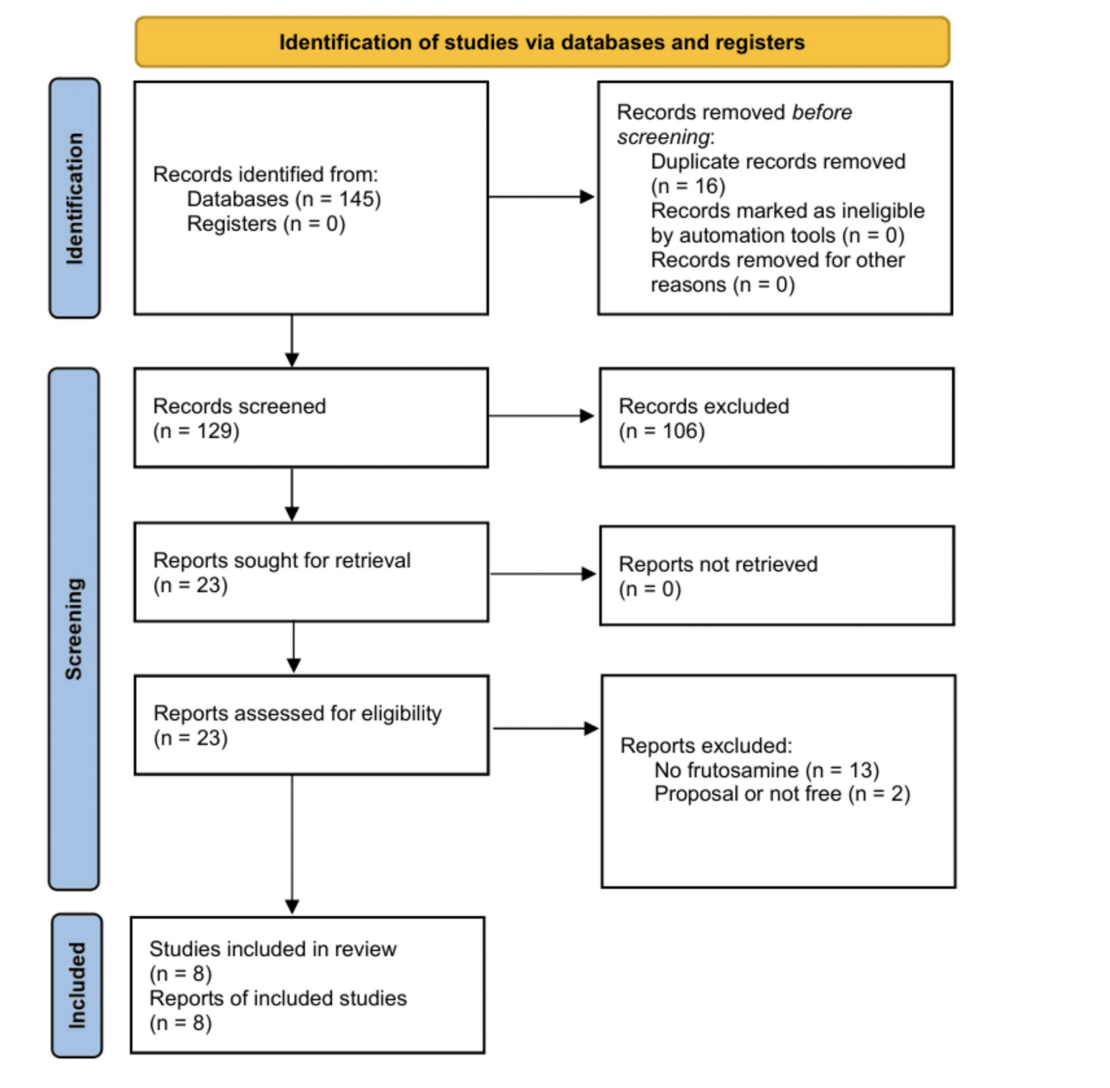
PRISMA flowchart PRISMA: Preferred Reporting Items for Systematic reviews and Meta-Analyses [14]

The database search identified 145 papers, of which 137 were excluded via the PRISMA flowchart (Figure 1).

The eight studies included in this review [15-22] and the results derived from them are discussed below.

Some of the included studies demonstrated that, in populations with the sickle cell trait, there is a correlation between fasting blood glucose and fructosamine, especially in areas with high prevalence of sickle cell disease (Table 2).

**Table 2:** The correlation between fructosamine and fasting glucose

Population	Correlation (Fasting glucose vs. Fructosamine)	Sample size	Reference
Area with high prevalence of sickle cell disease	r = 0.37	437	Nouya et al. [15]
Sickle cell trait	r = 0.83, p<0.001	821	Doumatey et al. [18]
Sickle cell trait	r = 0.39, p<0.01	821	Skinner et al. [19]

Furthermore, HbA1c and fructosamine were highly correlated in patients without sickle cell disease (Table 3).

**Table 3:** The correlation between HbA1c and fructosamine

HbA1c and fructosamine	Studies
r = 0.80, p<0.001	Doumatey et al. [18]
r2=0.61	Cohen et al. [17]

Doumatey et al. [18] showed that fructosamine levels were similar in individuals with sickle cell trait hemoglobin (287 µmol/L) and those with normal hemoglobin (275 µmol/l; p=0.11), indicating that the presence of sickle cell trait does not affect the normal formation or concentration of fructosamine.

Fructosamine and HbA1c were well-correlated in patients with and without diabetes (Table 4). 

**Table 4:** Correlation between fructosamine and HbA1c

Studies	Fructosamine ( µmol/L )	HbA1c (%)
Cohen et al. (non-diabetic population) [22]	240 +/- 19	5.2 +/- 0.4
Cohen et al. (diabetic population) [22]	264 +/- 97	8.5 +/- 3.0
Parrinello et al. (Black population) [21]	260 (236-285)	6.6 (6.2-7.2%)
Parrinello et al. (White population) [21]	247 (229-268)	6.2 (5.8-6.8)

Discussion

Many international guidelines recommend HbA1c for monitoring glycemic control in patients with diabetes. Despite these recommendations, there are ongoing concerns about the accuracy of HbA1c in populations with a high prevalence of sickle cell disease since sickle cell hemoglobin affects the accuracy of some HbA1c assays [15].

Fructosamine, on the other hand, is produced by the glycation of serum proteins and is not affected by red blood cell lifespan. In the Sub-Saharan Africa region, where sickle cell disease is very common, the concentration of serum fructosamine is significantly elevated in patients with diabetes compared to those with normoglycemia (p<0.0001) [16]. Hence, fructosamine levels could potentially replace HbA1c as a marker in patients with sickle cell disease and diabetes. Although the measurement of fructosamine (two to three weeks) and HbA1c (two to three months) reflect different periods, the comparison between the predicted values of HbA1c derived from fructosamine measurements and the actual HbA1c values indicates a significant correlation between the two metrics, with an r² value of 0.61 [17].

Though this study did not explicitly aim to evaluate the impact of sickle cell hemoglobin, it nonetheless demonstrated that HbA1c and fructosamine are strongly correlated in patients without sickle cell disease. This association is further supported by the findings of Doumatey et al. [18], who demonstrated that in individuals diagnosed with diabetes, fructosamine exhibited a strong correlation with HbA1c in those with normal hemoglobin (0.80; p<0.001). Additionally, research conducted by Skinner et al. [19] indicated a fairly significant correlation between fructosamine and HbA1c in both non-sickle cell control groups and those with sickle cell trait, yielding correlation coefficients of r=0.71 (p<0.0001) and r=0.61 (p<0.0001), respectively. This positive correlation within the sickle cell trait group can be attributed to the use of the Capillary 3-Tera device (Sebia, France) for HbA1c measurement, which demonstrated no interference from sickle cell hemoglobin [20].

A study found that individuals possessing the sickle cell trait and those with normal hemoglobin demonstrated comparable fructosamine levels (median: 287 vs. 275 µmol/L, p=0.11) [19]. This finding is noteworthy, particularly given the disparities observed in HbA1c levels between the two groups. Additionally, in patients with sickle cell disease who exhibit poorly managed diabetes, serum fructosamine concentrations were found to be significantly elevated [21]. This suggests that the presence of sickle cell hemoglobin does not compromise the accuracy of fructosamine measurement.

It is evident that fructosamine formation is not influenced by the type of hemoglobin present, with levels being comparable in individuals with sickle cell hemoglobin and those with normal hemoglobin. Additionally, there exists a strong correlation between fructosamine levels and random blood glucose in individuals with sickle cell disease. Thus, examining the relationships between fasting blood glucose and fructosamine, as well as between fructosamine and HbA1c, could help establish a standardized treatment protocol for patients with sickle cell disease who are also diabetic.

Table 3 shows a strong correlation between fructosamine and fasting glucose in a region with a high occurrence of sickle cell disease, specifically within a diverse Sub-Saharan African community, with a fairly robust correlation coefficient (r=0.37, p<0.0001) [15]. A similar finding was observed in a separate African population, demonstrating a notable fructosamine and fasting glucose correlation of 0.72 among individuals with normal hemoglobin and 0.83 among those with sickle cell trait (p<0.001) [19]. Furthermore, this positive finding was also reported in Senegalese individuals with diabetes and sickle cell trait hemoglobin, where a significant correlation was found between fructosamine and fasting blood glucose levels (r=0.39; p<0.01). This correlation was stronger than that in the control group (p<0.05) [20].

These results indicate that once an appropriate threshold for fructosamine is established, its serum levels can accurately reflect fasting blood glucose levels, thereby facilitating the identification of diabetes in individuals with sickle cell hemoglobin. Parrinello et al. [21] determined that a fructosamine level of 260 µmol/L corresponds to a fasting blood glucose level of 126 mg/dL, within a cohort of Black adults, with a p value of less than 0.001. In the analysis conducted by Cohen et al. [22], a comparison was made between fructosamine levels and HbA1c measurements. The study established that a fructosamine level of 240±19 µmol/L in individuals without diabetes corresponded to a HbA1c of 5.2±0.4%, while a value of 264±97 µmol/L in patients with diabetes corresponded to an HbA1c of 8.5±3.0% [22].

These findings are supported by both cross-sectional and longitudinal studies conducted by Malmström et al. [23] who reported that fructosamine has a 61% accuracy rate in diagnosing diabetes and a 97% rejection rate in diagnosing absence of diabetes, showing reasonable sensitivity and specificity. In their investigation, Parrinello et al. established the fasting blood glucose cut-off point as ≥126 mg/dL, in accordance with American Diabetes Association guidelines [11], which corresponded to fructosamine levels of 247 (229-268) µmol/L in White participants and 260 (236-285) µmol/L in Black participants (p<0.001) [21]. These values also corresponded to HbA1c levels of 6.2% (5.8-6.8) in White individuals and 6.6% (6.2-7.2) in Black individuals (p<0.001), indicating that fructosamine levels in the range of 240-260 µmol/L can be used in patients with sickle cell hemoglobin to identify those with diabetes.

Conversely, Doumatey et al. [18] provided a different recommendation, suggesting that a fructosamine cut-off of 309.5 µmol/L was suitable for detecting diabetic cases within their studied population. They assigned a significantly higher cut-off point when compared with the findings of Cohen et al. [22], especially for a HbA1c value equivalent to that of fructosamine.

A fructosamine level ranging from 240 to 310 µmol/L can be utilized as a target for diabetes management in individuals with sickle cell disease and diabetes. This range falls between the lower boundary of Cohen et al. [22] at 240 µmol/L and the higher cut-off point of 309.5 µmol/L proposed by Doumatey et al. [18]. It also encompasses the reference interval cited by Cohen et al. [22], who indicated a fructosamine value of 264±97 µmol/L in patients with diabetes, corresponding to an HbA1c of 8.5±3%.

In the context of diagnosing diabetes in individuals with sickle cell hemoglobin, fructosamine levels ranging from 240 to 260 µmol/L, can serve as a reliable indicator, with an average level of 260 µmol/L observed in the Black population. This assertion is further supported by the findings of Parrinello et al. [21], who determined that a fructosamine level of 260 µmol/L correlated with a fasting blood glucose of 126 mg/dL for the diagnosis of diabetes. This evaluation substantiates the relevance of fructosamine measurement for individuals presenting with either heterozygous or homozygous forms of sickle cell disease.

Nevertheless, to solidify this diagnosis based on fructosamine levels, additional research is required to establish an appropriate cut-off range (240-260 µmol/L) for this biomarker. In the interim, alternative diagnostic methods such as fasting blood glucose and oral glucose tolerance tests can be utilized.

Strengths and limitations

One of the primary strengths of this study lies in its exclusive focus on cohort studies, case-control studies, and cross-sectional studies, with the deliberate exclusion of case reports and editorial papers. A noted limitation of our research is the heterogeneity observed in the statistical testing among the included studies. Consequently, our investigation takes the form of a narrative synthesis for a systematic review, rather than a meta-analysis.

## Conclusions

Fructosamine is well correlated with fasting blood glucose in patients with sickle cell trait and populations with a high prevalence of sickle cell disease. It also demonstrates a strong correlation with HbA1c among individuals with the sickle cell trait and is notably elevated in patients with sickle cell disease and poorly controlled diabetes. These results imply that fructosamine could serve as a feasible tool for monitoring diabetes in individuals with sickle cell haemoglobin. Nevertheless, further research is required to establish an appropriate fructosamine cut-off point for the diagnosis of diabetes in patients with sickle cell disease.
